# A Systematic Review of Proton Therapy for the Management of Nasopharyngeal Cancer

**DOI:** 10.14338/IJPT-20-00082.1

**Published:** 2021-06-25

**Authors:** Anna Lee, Sarin Kitpanit, Marina Chilov, Johannes A. Langendijk, Jiade Lu, Nancy Y. Lee

**Affiliations:** 1 Department of Radiation Oncology, University of Texas MD Anderson Cancer Center, Houston, TX, USA; 2 Division of Radiation Oncology, Department of Radiology, Faculty of Medicine, Chulalongkorn University, King Chulalongkorn Memorial Hospital, Thai Red Cross Society, Bangkok, Thailand; 3 Medical Library, Memorial Sloan Kettering Cancer Center, New York, NY, USA; 4 Department of Radiation Oncology, University of Groningen, University Medical Center Groningen, Groningen, The Netherlands; 5 Department of Radiation Oncology, Shanghai Proton and Heavy Ion Center, Shanghai Cancer Hospital, Fudan University, Shanghai, China; 6 Department of Radiation Oncology, Memorial Sloan Kettering Cancer Center, New York, NY, USA

**Keywords:** nasopharyngeal cancer, proton therapy, systematic review

## Abstract

**Purpose:**

With improved technology, more patients with nasopharyngeal cancer (NPC) are receiving definitive treatment with proton therapy, which allows greater sparing of dose to normal tissues without compromising efficacy. As there is no randomized data, the purpose of this study was to systematically review the available literature on proton therapy in this setting, focusing on the toxicity endpoints.

**Materials and Methods:**

A systematic search using PRISMA (Preferred Reporting Items for Systematic Reviews and Meta-Analyses) guidelines was conducted in 5 databases: PubMed, Embase, SCOPUS, Web of Science, and the Cochrane Central Register of Controlled Trials. A total of 491 studies were found on the topic of NPC and proton therapy. Following independent study selection by 2 investigators, 9 studies were found to have sufficient focus and relevance to be incorporated into the systematic review.

**Results:**

All 9 studies were retrospective and examined only NPC patients except for one that also included paranasal sinus cancer. One study was a reirradiation study. Four studies used 3D or double scatter technique, while all others used intensity-modulated proton therapy. Oncologic outcomes were similar to intensity-modulated radiation therapy (IMRT) rates, with 2-year local and regional progression-free survival (LRFS) ranging from 84% to 100%, 2-year progression-free survival (PFS) ranging from 75% to 88.9%, and 2-year overall survival (OS) ranging from 88% to 95% in the up-front setting. Four comparison studies with IMRT found significantly lower feeding tube rates (20% versus 65%, *P* = .015; and 14% versus 85%, *P* < .001) with proton therapy as well as lower mucositis (G2 46% versus 70%, *P* = .019; and G3 11% versus 76%, *P* = .0002). All other acute and late effects were largely improved with proton therapy but not statistically significant.

**Conclusions:**

NPC patients receiving proton therapy maintain good outcomes with improved toxicity profile, likely due to sparing of dose to normal structures. Prospective studies are ongoing to better quantify the magnitude.

## Introduction

Nasopharyngeal carcinoma (NPC) comprises 0.6% of all cancers worldwide and is an endemic entity in Asia [1]. Owing to its anatomic location, the mainstay of treatment is a nonsurgical approach with radiation therapy alone for stage I and definitive chemoradiation for stage II and higher. Local control remains excellent at 90% [2–4], thus the focus has been on improving the toxicity profile from treatment as patients are living longer.

Prior to technologic advances, a survey study on the quality of life of patients with head and neck cancer found that NPC patients had the highest morbidity [5] likely due to the treatment fields encompassing salivary glands, pterygoid muscles, and temporomandibular joints. Another survey of NPC survivors found that xerostomia, hearing impairment, dysphagia, and trismus were the most common late sequelae following conventional radiation [6, 7]. Moreover, as compared to other patients with head and neck cancer, NPC patients are more likely to develop neurocognitive decline [8, 9]. Since then, intensity-modulated radiation therapy (IMRT) has been widely adopted following results from the Radiation Therapy Oncology Group (RTOG) phase II trial 0225 [2], which showed minimal toxicity yielding improved compliance rates throughout treatment, conferring benefit to local control.

While the increased conformality with IMRT allows full therapeutic dose to tumors near critical structures, there is still significant toxicity due to the entrance and exit dose in the beam paths [10]. Fortunately, proton therapy can further improve the therapeutic ratio as the radiation is able to penetrate to the depth of the target with little to no exit dose beyond it [11]. The technology with proton therapy has evolved where intensity-modulated proton therapy (IMPT) further reduces toxicity without compromising efficacy [12]. IMPT can now be delivered with spot-scanning, which scans the target without introducing scatter outside the beam path and further conforms the dose to target volumes by using different energies within the spots.

Planning comparison studies have shown dosimetric advantages of IMPT over IMRT with respect to delivering lower doses to organs at risk in NPC [13, 14]. Whether this translates into clinically meaningful endpoints showing reduction in acute and late toxicity is still unclear. To our knowledge, there are no prospective studies or published randomized trials evaluating the efficacy and toxicity of proton therapy in NPC even though proton therapy is rapidly increasing in use for this subsite. Our aim was therefore to perform a systematic review of the available literature to address the utility of proton therapy for adult patients with NPC, focusing on the toxicity profile.

## Materials and Methods

### Literature Search Strategy

This systematic review was conducted by using the Preferred Reporting Items for Systematic Reviews and Meta-Analyses (PRISMA) guidelines [15]. Literature searches were conducted on August 6, 2020, in 5 bibliographic databases by a medical librarian: PubMed, Embase (Embase.com), SCOPUS, Web of Science, and the Cochrane Central Register of Controlled Trials. The search strategy included components related to NPC and proton therapy. The inclusion criteria were broad initially to allow screening at the individual level. The search terms used were subject headings (MeSH, Emtree) and/or keywords in the title, abstract, and author keywords fields. Boolean operators ‘OR' and ‘AND' were used to combine the search terms and the search strategy components. Details of the search strategy are presented separately (see **Appendix**). The searches in all databases except for Cochrane CENTRAL were limited to the English language. Deduplication of search results from different databases was done with Covidence systematic review management software (Veritas Health Innovation, Melbourne, Australia).

### Study Selection

Based on the initial search, 491 studies were identified after duplicates were removed. Two reviewers (A.L. and S.K.) independently screened all studies, and 48 full-text studies were assessed for eligibility. The exclusion criteria were single case studies, review articles, abstracts, animal or phantom studies, pediatric studies, adenoid cystic carcinoma of the nasopharynx studies, and those that did not include toxicity endpoints. Studies including other heavy ion particles such as carbon and multiple head and neck subsites were also excluded, as endpoints were not reported separately for NPC patients receiving proton therapy. Comparison and reirradiation studies were included as well as those examining proton boost following photon therapy. No limit was imposed for the size of the cohort. All disagreements were resolved through discussion until consensus was reached. Thus, 9 original studies were incorporated into the systematic review. The selection process and inclusion of references in the systematic review is shown in the Figure.

**Figure.**
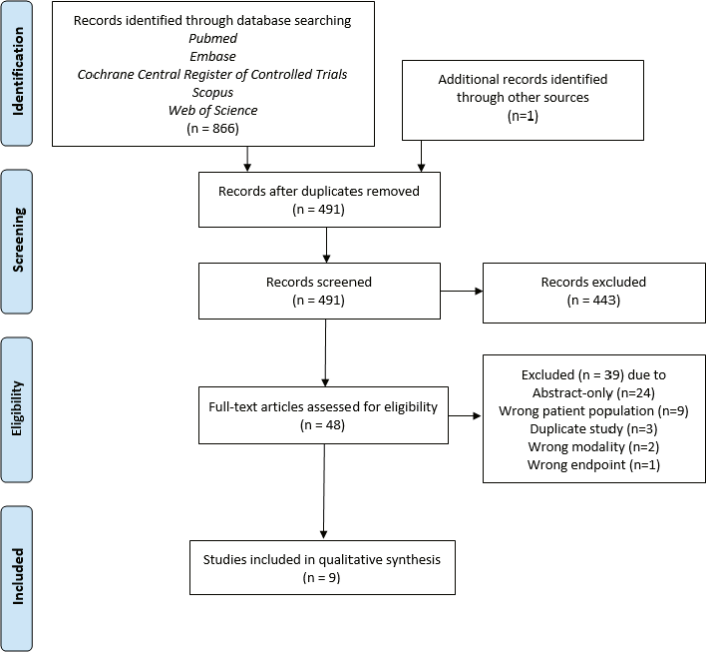
PRISMA diagram of the selection process and inclusion in the systematic review. Abbreviation: PRISMA, Preferred Reporting Items for Systematic Reviews and Meta-Analyses.

### Data Extraction

The following details were extracted from the included papers: authors, year of publication, type of study, number of patients, type of proton therapy, prescribed dose, number of fractions, systemic therapy (neoadjuvant, concurrent, adjuvant), stage, and follow-up period. The variations found in units of dose were kept as reported. Acute and late toxicity rates were the primary outcomes of interest, while local and regional progression-free survival (LRFS), progression-free survival (PFS), and overall survival (OS) were secondary endpoints. Any missing data were indicated as such.

## Results

As shown in the Figure, 9 total studies met criteria for data extraction, which included a total of 369 patients with a median follow-up of 25 months (range, 10–98 months). Details of the studies included in this review are provided in Table 1. All studies were of NPC patients except for one that also included patients treated for paranasal sinus primaries [16]. Four studies originated from the United States, 2 from Italy, 1 from France, 1 from the Czech Republic, and 1 from South Korea. All had at least 10 patients in the cohort and were entirely retrospective. Four studies were matched where patients receiving proton therapy were compared to patients receiving photon therapy [16, 17, 21, 23]. Three used a mixed-beam approach with photons followed by a proton therapy boost [19, 21, 23,]. Four of the 9 studies used 3D or double scatter while all others used IMPT. Prescribed doses ranged from 68.4 to 78 GyE except for 1 study from Italy examining reirradiation outcomes for recurrent NPC where prescribed dose was 60 GyE [20]. Concurrent chemotherapy was given in all 9 studies; 7 administered neoadjuvant systemic treatment and 4 gave adjuvant chemotherapy as well. Median follow-up for up-front radiation studies was 25.8 months and ranged from 21 to 98 months. With respect to oncologic endpoints, all studies except 1 reported either LRFS, PFS, OS, or a combination. As expected, outcomes were similar to IMRT rates with 2-year LRFS ranging from 84% to 100%, 2-year PFS ranging from 75% to 88.9%, and 2-year OS ranging from 88% to 95%. For the reirradiation study, the reported rate for 2-year LRFS was 72.9% and for OS, 59.3% [20].

**Table 1.** NPC and proton therapy study characteristics ordered by year published.

**Study (year; country)**	**Type**	**N**	**Modality**	**Prescribed dose**	**No. fraction**	**Systemic therapy**			**Stage**				**Median FU, mo**	**Outcomes at 2 y**		
						% NACT	% Con	% Adj	T1-2	T3-4	N0-1	N2-3		LRFS	PFS	OS
Holliday et al (2015; USA) [17]	Retrospective, matched	10 20	IMPT IMRT	70 GyE 70 Gy	33–35 33–35	80 85	100 90	10 0	60 55	40 45	40 35	60 65	21.6 25.8	N = 10^a^ N = 19^a^		N = 9^a^ N = 19^a^
Lewis et al (2016; USA) [18]	Retrospective	10	IMPT	70 GyE	33–35	77.8	100	22.2	66.6	33.3	44.4	55.6	24.5	100	88.9	88.9
McDonald et al (2016; USA) [16]^b^	Retrospective, matched	26 (15 NPC) 14 (2 NPC)	IMRT 3D-PT	71.4 Gy 71.8 GyE		0 21.4	88.5 50	0 14.2	19.2 7.1	80.8 92.9	57.7 78.6	42.3 21.4				
Beddok et al (2019; France) [19]	Retrospective	17	Mixed-beam EBRT^c^/ DS-PT	70–78 GyE	35–39	65	100		0	100	100	0	98	94	88	88
Dionisi et al (2019; Italy) [20]	Retrospective	17 rNPC	3D-PT	60 GyE (30.6–66)	1.8–2 Gy/Fx	11.8	47	0	5.9	88.2	0	0	10	72.9		59.3
Park et al (2019; Korea) [21]	Retrospective	63 35	IMRT Mixed-beam IMRT/IMPT	68.4 GyE 68.4 GyE	30 30	0 0	100 100	0 0	Stage: II 12.7%, III 36.5%, IV 50.8% Stage: II 45.7%, III 22.9%, IV 31.4%					81 (1 y) 87.1 (1 y)	
Sanford et al (2019; USA) [22]	Retrospective	12 61	IMRT DS-PT	70 Gy 70 GyE		29	92	38	28	72			90 90	96	85	92
Alterio et al (2020; Italy) [23]	Retrospective	17 27	IMRT Mixed-beam IMRT/IMPT	69.96–70 Gy 70–74 GyE	33–35 35–37	15 (88.2) 16 (59.3)	16 (94.1) 27 (100)		0 0	17 (100) 27 (100)	8 (47.1) 12 (44.4)	9 (52.9) 15 (55.6)	51 25	89 94	69 76	N = 15^a^ N = 22^a^
Jiri et al (2020; Czech Republic) [24]	Retrospective	40	IMPT	70–76 GyE	35–38		34 (85)		19 (44.5)	24 (56.5)	14 (32.5)	29 (67.5)	24	84	75	80

**Abbreviations:** NPC, nasopharyngeal cancer; GyE, Gray equivalent; FU, follow-up; NACT, neoadjuvant chemotherapy; Con, concurrent chemotherapy; Adj, adjuvant chemotherapy; LRFS, local and regional progression-free survival; PFS, progression-free survival; OS, overall survival; IMPT, intensity-modulated proton therapy; -, no information; IMRT, intensity-modulated radiation therapy; 3D-PT, 3-dimensional conformal proton therapy; EBRT, external beam radiation therapy; DS-PT, double-scattering proton therapy; rNPC, recurrent nasopharyngeal cancer; 3DCRT, 3-dimensional conformal radiation therapy.

a At the time of last follow-up.

b The study included nasopharyngeal and paranasal sinus sites. No separate outcome for each site was reported. The values shown in the table therefore represent the entire cohort.

c EBRT = 3DCRT or IMRT.

### Toxicities

All studies reported various acute toxicities but there was heterogeneity in the reporting of the data. For example, 1 study did not clearly correlate the acute toxicity with the treatment modality (proton versus IMRT) [22]. The Common Terminology Criteria for Adverse Events was used for grading of side effects except for 1 study [24], which used the RTOG toxicity grading system. Of the 4 matched studies, 2 compared feeding tube rates and found significantly improved rates in the proton cohort (20% versus 65%, *P* = .015 [17] and 14% versus 85%, *P* < .001 [16]). Two other matched studies found that of the reported toxicities, higher-grade mucositis was significantly improved in the proton cohort (G2 46% versus 70%, *P* = .019 [21] and G3 11% versus 76%, *P* = .0002 [23]). All other acute toxicities were not significantly different between modalities in these 2 studies. Details on reported acute toxicities can be found in Table 2.

**Table 2.** NPC and proton therapy acute toxicities ordered by year published.

**Study (year)**	**Proton technique**	**IMRT, N (%)**					**Proton, N (%)**						***P*** **value**
		**Dermatitis**	**Mucositis**	**Wt loss**	**Feeding tube/ swallowing**	**Xerostomia**	**Dermatitis**	**Mucositis**	**Wt loss**	**Feeding tube/ swallowing**	**Xerostomia**	**Others**	
Holliday et al (2015) [17]	IMPT	G1+ 30 (100)		%wt loss = 5.7	Overall 13 (65)^a,b^		G1 1 (10) G2 4 (40) G3 4 (40)		%wt loss = 7.6	Overall 2 (20)^a,b^			^a^Feeding tube .015
Lewis et al (2016) [18]	IMPT						G2 5 (50) G3 4 (40)	G2 8 (80) G3 1 (10)	%wt loss = 6 G1 5 (50) G2 3 (30)	Overall 3 (30)^b^			
McDonald et al (2016) [16]	3D-PT				Overall 22 (84.6)^a^					Overall 2 (14.3)^a^			^a^Feeding tube <.001
Beddok et al (2019) [19]	Mixed-beam EBRT^c^/ DS-PT									G3 1 (5.9)		Middle ear inflammation 6 (35)	
Dionisi et al (2019) [20]	3D-PT						G1 3 (17.6)	G1 4 (23.5), G2 3 (17.6)				G1 hearing 1 (5.9) G1 fatigue 2 (11.7) G1 CN disorder 1 (5.9) G1 pain 3 (17.6) G2 pain 1 (5.9)	
Park et al (2019) [21]	Mixed-beam IMRT/ IMPT	G3+ 2 (3.2)	G2+ 44 (69.8)^a^				G3+ 5 (14.3)	G2+ 16 (45.7)^a^				Analgesic use: G2 54 (IMRT) vs 37.1% (mixed beam)	^a^Mucositis .019
Alterio et al (2020) [23]	Mixed-beam IMRT/IMPT	G1 8 (47.1) G2 9 (52.9)	G1 2 (11.8) G2 2 (11.8) G3 13 (76.4)^a^	G1 9 (52.9) G2 6 (35.3)	G1 7 (41.2) G2 5 (29.4) G3 4 (23.5)	G1 10 (58.8) G2 6 (35.3)	G1 9 (33.3) G2 15 (55.6)	G1 11 (40.8) G2 13 (48.1) G3 3 (11.1)^a^	G1 14 (51.8) G2 5 (18.5)	G1 11 (40.8) G2 8 (29.6) G3 4 (14.8)	G1 19 (70.4) G2 2 (7.4)		^a^Mucositis .0002
Jiri et al (2020) [24]^d^	IMPT						G1 8 (18.6) G2 29 (67.4) G3 6 (14)	G1 11 (25.6) G2 28 (65.1) G3 3 (7)	Deviation from baseline: >15%: 13 (30.2), 5%–15%: 26 (60.5), <5%: 4 (9.3)	G1 12 (27.9) G2 18 (41.9) G3 4 (9.3)			

**Abbreviations:** NPC, nasopharyngeal cancer; IMRT, intensity-modulated radiation therapy; Wt, weight; IMPT, intensity-modulated proton therapy; G, toxicity grade; -, no information; 3D-PT, 3-dimensional conformal proton therapy; EBRT, external beam radiation therapy; DS-PT, double-scattering proton therapy; CN, cranial nerve; RTOG, Radiation Therapy Oncology Group.

a Statistically significant.

b Placement during or after treatment.

c EBRT=3DCRT or IMRT.

d Toxicity evaluation using RTOG scale.

With respect to late effects, the most frequently reported were xerostomia and hearing loss. One study comparing a mixed-beam approach with IMRT/IMPT compared to IMRT alone found the proton group trended toward less soft tissue fibrosis (*P* = .07) [23]. One matched study where gastrostomy tube insertion was the primary endpoint found 2 cases of G1 temporal lobe necrosis (TLN) in the IMRT group and 1 case each of G1 TLN and G3 TLN in the proton group [17]. One study using a mixed-beam approach reported 1 grade-5 necrosis–induced nasopharyngeal bleed and upon further review, the patient had T4 disease with significant comorbidities including human immunodeficiency virus and hepatitis B and C coinfections [19]. Furthermore this study prescribed doses up to 78 GyE. The reirradiation study reported overall grade 3 to 5 toxicities of 23.5% [20]. Details on reported late toxicities can be found in Table 3.

**Table 3.** NPC and proton therapy late toxicities ordered by year published.

**Study (year)**	**IMRT, N (%)**						**Proton, N (%)**				
	**Proton technique**	**Xerostomia**	**Feeding tube/ swallowing**	**Hearing**	**TLN**	**Others**	**Xerostomia**	**Feeding tube/ swallowing**	**Hearing**	**TLN**	**Others**
Holliday et al (2015) [17]	IMPT		3 (15)^a^	G3 2 (10)	G1 2 (10)		3 (30)	0^a^		G1 1(10) G3 1(10)	
Lewis et al (2016) [18]	IMPT						G1 6 (60) G2 2 (20)				
Beddok et al (2019) [19]	Mixed-beam EBRT^b^/ DS-PT						12 (70.6)		Overall 9 (52.9) G3 4 (23.5)	Overall 6 (35.3) G2+ 1 (5.9)	Gr5 necrosis-induced nasopharyngeal bleeding 1 (5.9)
Dionisi et al (2019) [20]	3D-PT							G2 1 (5.9) G3 1 (5.9)	G3 3 (17.6)		G2 trismus 1 (5.9), G2 soft tissue necrosis 2 (11.8), G2 osteonecrosis 1 (5.9)
Sanford et al (2019) [22]	DS-PT			G3 1 (8.3)					G3 4 (7)	G3 1 (1.6)	G3 CN deficit 1 (1.6), no G2 xerostomia in both modalities
Alterio et al (2020) [23]	Mixed-beam IMRT /IMPT	G0-1 11 (68.8) G2 5 (31.3)	G0-1 16 (100)	G0-1 15 (93.8) G2 1 (6.3)	G0-1 16 (100)		G0-1 16 (61.5) G2 10 (38.5)	G0-1 26 (100)	G0-1 25 (96.2) G2 1 (3.8)	G0-1 26 (100)	Trend to less soft tissue fibrosis (*P* = .07)
Jiri et al (2020) [24]^c^	IMPT						G2 3 (7)	G2 2 (5)	G2 3 (7) G3 0	G3+ 1 (2)	

**Abbreviations:** NPC, nasopharyngeal cancer; IMRT, intensity-modulated radiation therapy; TLN, temporal lobe necrosis; IMPT, intensity-modulated proton therapy; -, no information; G, toxicity grade; EBRT, external beam radiation therapy; DS-PT, double scattering proton therapy; 3D-PT, 3-dimensional conformal proton therapy; CN, cranial nerve; 3DCRT, 3-dimensional conformal radiation therapy; RTOG, Radiation Therapy Oncology Group.

a Swallowing dysfunction evaluated by a speech pathologist of a modified barium swallow.

b EBRT = 3DCRT or IMRT.

c Toxicity evaluation using RTOG scale.

## Discussion

This study provides a current and systematic review focusing on outcomes for NPC patients treated with proton therapy. We found planning studies were common but those focusing on actual toxicity rates are relatively rare, thus 9 studies were formally included in this review. Encouragingly, the studies were diverse in country of origin, indicating that the use of proton therapy in this subsite is increasingly adopted worldwide.

The benefit of proton therapy with respect to minimizing dose to normal structures has been clearly elucidated in dosimetric studies. Namely, Jacobi et al [25] used modern normal tissue complication probability (NTCP) modeling for acute toxicities to compare IMRT to IMPT. They found IMPT was able to maintain target coverage while reducing NTCP values and identified patients with upper head and neck tumors as gaining the most benefit with IMPT, particularly for swallowing-related side effects. Another planning comparison study of NPC patients found that the volume of mucosa and esophagus receiving ≥20 Gy and ≥30 Gy was significantly lower with IMPT than with helical tomotherapy and this was the case for most other organs at risk assessed [14]. This appeared to translate into clinically meaningful endpoints as shown in 2 comparison studies [16, 17], resulting in significantly lower feeding tube rates with proton therapy, even when using 2D conformal techniques. McDonald et al [16] also found a significantly lower opioid pain medication requirement with proton therapy in its cohort of patients with NPC and paranasal sinus cancer (odds ratio 0.09; 95% confidence interval, 0.01–0.57; *P* = .006).

Another acute toxicity that was notably improved was higher-grade mucositis. In the IMRT era, RTOG 0225 reported 29.4% for mucositis inhibiting oral intake [2], Cao et al [26] reported 21% for grade 3 mucositis in T4 NPC patients, and Songthong et al [27] reported 15% for grade 3 or higher mucositis. In the current series of proton studies, grade 3 or higher mucositis ranged from 7% to 11.1%. Two of these studies compared a mixed-beam approach to IMRT and still found significantly lower rates for higher-grade oral mucositis [21, 23]. The reirradiation study from our Italian colleagues [20] reported a rate of 17.6% for grade 2 mucositis among their 17 patients, of which half received concurrent chemotherapy. As supported by Lewis et al [18], proton therapy minimizes dose to the anterior cavity, which translates into lower rates of higher-grade oral mucositis toward the end of treatment, even offering substantial benefit when used as a boost over IMRT alone.

Another advantage of proton therapy is the decreased integral dose [28], which offers the potential advantage of lowering the incidence of radiation-induced tumors and allows room for reirradiation as patients are living longer with improved outcomes. Similarly, late effects also become a concern. The median follow-up for the up-front radiation studies was 25.8 months, which is not considerably long in a disease site with 90% local control.

In the studies reporting xerostomia, the mixed-beam approaches generally had higher rates of grade 2 (20%–38.5%) versus IMPT alone (7%), but grade 1 rates in IMPT studies (60%–61.5%) were comparable to those of IMRT studies [29, 30]. Planning studies have shown lower mean doses to the parotids and submandibular glands [13, 14], which should theoretically translate into lower xerostomia rates. Thus, the variation in rates may be related to physician grading, heterogeneity of the patients in the cohorts, and quality of the treatment planning at different centers.

Other notable late toxicities included hearing loss, which ranged widely from grade 2 at 3.8% [23] to grade 4 at 23.5% [19]. These were from mixed-beam studies and were similar to published IMRT studies at 25% from a large retrospective investigation of 869 patients and 13% for grade 2+ auditory toxicity from RTOG 0225 [2]. Even though the mixed-beam studies were published contemporaneously, some of the patients included were treated more commonly with 3D conformal techniques in the early 2000s, which may account for higher than expected rates. A purely IMPT study with median follow-up of 2 years reported lower rates with grade 2 hearing impairment at 7% and no grade 3 hearing changes [24].

Temporal lobe necrosis rates are comparable for patients treated with mixed beam and with pure IMRT and IMPT. In the mixed-beam studies, Alterio et al [23] reported no clinically significant central nervous system (CNS) necrosis (100% grade 0-1), and Beddok et al [19] found only 1 case of grade 2 TLN with all others having grade 0 to 1 necrosis. The TLN rates were also low among patients receiving only proton therapy with 1 case of grade 3 TLN in each reported study by Holliday et al [17], Jiri et al [24], and Sanford et al [22]. Of note, IMRT overall TLN rates of up to 15% have been reported with longer follow-up (beyond 5 years) [31, 32]; however, these were not graded, thus highlighting the lack of consistency in the reporting of rates. Literature suggests earlier onset of TLN following treatment with proton therapy compared to IMRT [33], which may be due to lack of conformality at the high-dose region, particularly with older proton techniques as used in the study by Beddok and colleagues [19]. Furthermore, the dose delivered may be higher at the distal edge, as the RBE can be higher than 1.1. Finally, MRI is a common modality to assess NPC patients, thus its use in monitoring tumor response and treatment-related CNS changes may inevitably detect changes that are subtle.

It is notable that all the studies in this review are retrospective in nature. Currently, a phase II trial from the Shanghai Proton and Heavy Ion Center (NCT04528394) is recruiting NPC patients to be randomly assigned to photon followed by carbon boost versus proton followed by carbon boost with grade 2 xerostomia at 6 months as the primary endpoint [34]. A completed phase II trial (NCT00592501) evaluating proton boost for NPC patients with concurrent chemotherapy evaluated acute toxicities, quality of life, and treatment compliance [35]. Long-term results are pending. Finally, HN001 (NCT02135042) evaluating postchemoradiation adjuvant therapy based on plasma Epstein-Barr virus DNA levels allows proton therapy, thus subgroup analysis is anticipated to assess the benefit in this cohort [36] (Table 4).

**Table 4.** Ongoing clinical studies focusing on NPC population registered on www.clinicaltrials.gov as of October 2020.

**ClinicalTrials.gov identifier**	**Phase**	**Study**	**Institute**	**Arm 1**	**Arm 2**	**Primary endpoint**	**Estimated primary completion**
NCT04528394 [34]	II, recruiting	A Randomized Phase II Trial Evaluating Toxicity and Efficacy Between Proton and Photon for Nasopharyngeal Carcinoma	Shanghai Proton and Heavy Ion Center Shanghai, Shanghai, China	Photon + carbon	Proton + carbon	Grade 2 xerostomia at 6 mo	June 2021
NCT00592501 [35]	II, completed	A Phase II Study of Proton Radiotherapy With Chemotherapy for Nasopharyngeal Carcinoma	MGH, Boston, Massachusetts, USA	Photon + proton with cisplatin/5-fluorouracil		Acute toxicities, QOLs, treatment compliance	Completed
NCT02135042^a^ [36]	II/III, recruiting	Randomized Phase II and Phase III Studies of Individualized Treatment for Nasopharyngeal Carcinoma Based on Biomarker Epstein Barr Virus (EBV) Deoxyribonucleic Acid (DNA)	Multi-institutional study, USA	Cisplatin/5-fluorouracil after chemoradiation^a, b^ Cisplatin/5-fluorouracil after chemoradiation^a, c^	Gemcitabine/paclitaxel after chemoradiation^a, b^ Observation after chemoradiation^a, c^	Progression-free survival Overall survival	July 2021

**Abbreviations:** NPC, nasopharyngeal cancer; MGH, Massachusetts General Hospital; QOLs, quality-of-life outcomes; EBV, Epstein-Barr virus.

a The trial allows both IMRT and IMPT during chemoradiation.

b Phase II, detectable plasma EBV DNA.

c Phase III, undetectable plasma EBV DNA.

The limitations of this review include the small number of studies, which is not unexpected as the implementation of this technology is still on the rise. Furthermore, the heterogeneity of the included studies with respect to proton technique, utilization of systemic therapy, and inclusion of proton boost following photon-based radiation makes aggregated interpretation of the data difficult. Missing data also limited our ability to perform quantitative pooled analyses. The relatively short follow-up also limited our understanding of all late effects that could manifest. Still, this review provides a snapshot of the toxicity profile, which will be further elucidated with longer follow-up.

In conclusion, this systematic review confirms that NPC patients receiving proton therapy have outcomes comparable to those of photon-based radiation with a largely improved toxicity profile, likely due to sparing of dose to normal structures. We await results from prospective studies to better quantify the magnitude of benefit.

## Appendix

### Literature Search Strategies

#### PubMed

(“Nasopharyngeal Neoplasms”[Mesh] OR “Pharyngeal Neoplasms”[Mesh:NoExp] OR “Nasopharynx”[Mesh] OR “Pharynx”[Mesh:NoExp] OR nasopharynx[tiab] OR naso-pharynx[tiab] OR nasopharyngeal[tiab] OR naso-pharyngeal[tiab] OR rhinopharynx[tiab] OR rhino-pharynx[tiab] OR rhinopharyngeal[tiab] OR rhino-pharyngeal[tiab] OR choana[tiab] OR choanae[tiab] OR choanal[tiab]) AND ((“Proton Therapy”[Mesh] OR “Heavy Ion Radiotherapy”[Mesh:NoExp] OR proton therapy[tiab] OR proton treatment[tiab] OR IMPT[tiab] OR PBT[tiab] OR particle therapy[tiab] OR particle treatment[tiab]) OR ((“Protons”[Mesh] OR proton[tiab] OR protons[tiab] OR particle[tiab]) AND (“Radiotherapy”[Mesh] OR “Radiotherapy Planning, Computer-Assisted”[Mesh] OR “Radiometry”[Mesh] OR “radiotherapy”[Subheading] OR radiotherapy[tiab] OR radio-therapy[tiab] OR chemoradiation[tiab] OR chemoradiotherapy[tiab] OR radio-chemotherapy[tiab] OR radiochemotherapy[tiab] OR radiation[tiab] OR reirradiation[tiab] OR irradiation[tiab] OR irradiate[tiab] OR irradiated[tiab] OR irradiating[tiab] OR dosimetry[tiab] OR dosimetric[tiab] OR beam[tiab] OR beams[tiab]))) AND “English”[la]

#### Embase

(‘nasopharynx tumor'/exp OR ‘nasopharynx'/exp OR ‘pharynx tumor'/de OR ‘nasopharynx':ti,ab,kw OR ‘naso pharynx':ti,ab,kw OR ‘nasopharyngeal':ti,ab,kw OR ‘naso pharyngeal':ti,ab,kw OR ‘rhinopharynx':ti,ab,kw OR ‘rhino-pharynx':ti,ab,kw OR ‘rhinopharyngeal':ti,ab,kw OR ‘rhino-pharyngeal':ti,ab,kw OR ‘choana':ti,ab,kw OR ‘choanae':ti,ab,kw OR ‘choanal':ti,ab,kw) AND (‘proton therapy'/exp OR ‘particle therapy'/de OR ‘proton therapy system'/exp OR ‘proton radiation'/exp OR ‘proton therapy':ti,ab,kw OR ‘proton treatment':ti,ab,kw OR ‘impt':ti,ab,kw OR ‘pbt':ti,ab,kw OR ‘particle therapy':ti,ab,kw OR ‘particle treatment':ti,ab,kw OR ((‘proton'/exp OR ‘proton':ti,ab,kw OR ‘protons':ti,ab,kw OR ‘particle':ti,ab,kw) AND (‘radiotherapy'/exp OR ‘radiotherapy planning'/exp OR ‘radiotherapy planning system'/exp OR ‘radiation measurement'/exp OR ‘radiotherapy':lnk OR ‘radiotherapy':ti,ab,kw OR ‘radio-therapy':ti,ab,kw OR ‘chemoradiation':ti,ab,kw OR ‘chemoradiotherapy':ti,ab,kw OR ‘radio-chemotherapy':ti,ab,kw OR ‘radiochemotherapy':ti,ab,kw OR ‘radiation':ti,ab,kw OR ‘reirradiation':ti,ab,kw OR ‘irradiation':ti,ab,kw OR ‘irradiate':ti,ab,kw OR ‘irradiated':ti,ab,kw OR ‘irradiating':ti,ab,kw OR ‘dosimetry':ti,ab,kw OR ‘dosimetrical':ti,ab,kw OR ‘dosimetrically':ti,ab,kw OR ‘dosimetric':ti,ab,kw OR ‘beam':ti,ab,kw OR ‘beams':ti,ab,kw))) AND [english]/lim

#### Cochrane Central Register of Controlled Trials

##### Title Abstract Keyword

( “nasopharynx” OR “naso-pharynx” OR “nasopharyngeal” OR “naso-pharyngeal” OR “rhinopharynx” OR “rhino-pharynx” OR “rhinopharyngeal” OR “rhino-pharyngeal” OR “choana” OR “choanae” OR “choanal” ) AND ( ( “proton therapy” OR “proton treatment” OR “IMPT” OR “PBT” OR “particle therapy” OR “particle treatment” ) OR ( ( “proton” OR “protons” OR “particle” ) AND ( “radiotherapy” OR “radio-therapy” OR “chemoradiation” OR “chemoradiotherapy” OR “radio-chemotherapy” OR “radiochemotherapy” OR “radiation” OR “reirradiation” OR “irradiation” OR “irradiate” OR “irradiated” OR “irradiating” OR “dosimetry” OR “dosimetrical” OR “dosimetrically” OR “dosimetric” OR “beam” OR “beams” ) ) )

#### Scopus

( TITLE-ABS-KEY ( “nasopharynx” OR “naso-pharynx” OR “nasopharyngeal” OR “naso-pharyngeal” OR “rhinopharynx” OR “rhino-pharynx” OR “rhinopharyngeal” OR “rhino-pharyngeal” OR “choana” OR “choanae” OR “choanal” ) ) AND ( ( TITLE-ABS-KEY ( “proton therapy” OR “proton treatment” OR “IMPT” OR “PBT” OR “particle therapy” OR “particle treatment” ) ) OR ( TITLE-ABS-KEY ( “proton” OR “protons” OR “particle” ) AND TITLE-ABS-KEY ( “radiotherapy” OR “radio-therapy” OR “chemoradiation” OR “chemoradiotherapy” OR “radio-chemotherapy” OR “radiochemotherapy” OR “radiation” OR “reirradiation” OR “irradiation” OR “irradiate” OR “irradiated” OR “irradiating” OR “dosimetry” OR “dosimetrical” OR “dosimetrically” OR “dosimetric” OR “beam” OR “beams” ) ) ) AND ( LIMIT-TO ( LANGUAGE , “English” ) )

#### Web of Science

##### Topic Search

( “nasopharynx” OR “naso-pharynx” OR “nasopharyngeal” OR “naso-pharyngeal” OR “rhinopharynx” OR “rhino-pharynx” OR “rhinopharyngeal” OR “rhino-pharyngeal” OR “choana” OR “choanae” OR “choanal” ) AND ( ( “proton therapy” OR “proton treatment” OR “IMPT” OR “PBT” OR “particle therapy” OR “particle treatment” ) OR ( ( “proton” OR “protons” OR “particle” ) AND ( “radiotherapy” OR “radio-therapy” OR “chemoradiation” OR “chemoradiotherapy” OR “radio-chemotherapy” OR “radiochemotherapy” OR “radiation” OR “reirradiation” OR “irradiation” OR “irradiate” OR “irradiated” OR “irradiating” OR “dosimetry” OR “dosimetrical” OR “dosimetrically” OR “dosimetric” OR “beam” OR “beams” ) ) )

Refined by: LANGUAGES: ( ENGLISH )

Timespan: All years. Indexes: SCI-EXPANDED, SSCI, A&HCI, CPCI-S, CPCI-SSH, BKCI-S, BKCI-SSH, ESCI, CCR-EXPANDED, IC.
